# Up-to-date evidence on image-guided thermal ablation for metastatic lung tumors: a review

**DOI:** 10.1007/s11604-022-01302-0

**Published:** 2022-07-02

**Authors:** Yusuke Matsui, Koji Tomita, Mayu Uka, Noriyuki Umakoshi, Takahiro Kawabata, Kazuaki Munetomo, Shoma Nagata, Toshihiro Iguchi, Takao Hiraki

**Affiliations:** 1 Department of Radiology, Faculty of Medicine, Dentistry and Pharmaceutical Sciences, Okayama University, 2-5-1 Shikata-cho, Kita-ku, Okayama, 700-8558 Japan; 2 Department of Radiology, Okayama University Hospital, Okayama, Japan; 3 Department of Radiological Technology, Faculty of Health Sciences, Okayama University, Okayama, Japan

**Keywords:** Ablation, Lung, Pulmonary, Metastasis

## Abstract

The aim of this review was to summarize the latest evidence on image-guided thermal ablation therapies for lung metastases. PubMed was used to search for relevant articles that reported the oncological outcomes of thermal ablation for metastatic lung tumors, and those published in 2010 or later were selected for review. Ablative therapies were applied for lung metastases from various types of primary tumors, but most commonly colorectal ones. Radiofrequency ablation (RFA) was the most evaluated technique, followed by microwave ablation (MWA). The local control rates of ablative therapies were generally favorable, approximately 80–90% in many studies. Representative studies demonstrated promising overall survival rates of approximately 50% or higher 5 years after ablation for lung metastases from colorectal cancer or mixed types of primary tumors. Nevertheless, the survival outcomes varied depending on the type of primary tumor and background factors of patients such as other metastases and comorbidities. Several studies had aimed to compare the outcomes of various ablative therapies such as RFA, MWA, and cryoablation; however, conclusive data are not yet available to determine the most appropriate ablation modality for lung metastases. Further data accumulation is needed, especially for long-term outcomes and comparisons with other therapies.

## Introduction

The lung is a frequent site for various types of malignant tumors to metastasize [1, 2]. Local therapies such as surgery, radiotherapy, and thermal ablation may be beneficial for patients with lung metastases, especially when complete eradication of all metastases is anticipated [3]. Because of their minimal invasiveness and repeatable nature, image-guided thermal ablation therapies such as radiofrequency ablation (RFA), microwave ablation (MWA), and cryoablation (CA) have been used to treat lung tumors, particularly in patients with contraindications to surgery. The Society of Interventional Radiology deems image-guided ablation an acceptable treatment option for patients with metastatic lung disease in their multidisciplinary position statement [4]. The Cardiovascular and Interventional Radiological Society of Europe also describes the role of thermal ablation for lung metastases in their Standards of Practice document [3]. The present article provides an overview of the latest evidence on the oncological outcomes of image-guided thermal ablation therapies for lung metastases by reviewing the literature published since 2010.

### Literature search

PubMed was used on April 19, 2022 to search for articles that included the following terms in their titles: ("lung” OR "pulmonary") AND ("metastasis" OR "metastases" OR "metastatic") AND ("ablation” OR "ablative"). The initial search identified 247 articles, of which 188 published since 2010 (considered the most recent articles) were selected. Articles were further screened based on their titles, abstracts, and text for the following criteria: (i) English language articles, (ii) original clinical studies or meta-analyses, and (iii) studies focusing on the oncological outcomes of thermal ablation for metastatic lung tumors that reported specific survival data such as overall survival (OS). We excluded small series with fewer than 10 patients and studies in which lung ablation was used for palliative purposes. Furthermore, studies with mixed study populations of primary and metastatic lung tumors, which precluded the evaluation of discrete data on lung metastases, were excluded. When multiple studies in the same institution had considerable overlapping in terms of subjects, the study with a larger sample size was selected for review. Screened articles were scrutinized further to include relevant articles from their reference lists in the final review.

### Ablative therapies for lung metastases: an overview of recent literature

Among the reviewed studies, large-scale ones tended to include patients with mixed types of primary tumors. In the studies that included each individual type of primary tumor, lung metastasis from colorectal cancer (CRC) was focused on the most, followed by that from sarcoma. RFA was the most commonly used technique, followed by MWA. Long-term outcomes were mainly available for RFA. Most studies on MWA, CA, and laser interstitial thermal therapy (LITT) were published from 2016 onward. Some studies aimed to compare the outcomes of multiple types of thermal ablation therapies, as described below.

### Outcomes in studies that included mixed types of primary tumors

Table 1 shows the results of studies that included mixed types of primary tumors [5–16]. Although those studies included various primary tumors, most predominantly included CRC, with only one focusing on non-CRC lung metastases [9]. The largest study was a meta-analysis recently published by Nguyenhuy et al. that analyzed, from 23 studies, 1804 patients who underwent RFA, MWA, or CA; notably, this meta-analysis included eight Japanese studies [5]. The 1-, 2-, 3-, 4-, and 5-year OS/progression-free survival (PFS) rates in the meta-analysis were 92.3%/53.1%, 80.0%/34.8%, 67.9%/25.4%, 58.8%/22.2%, and 50.7%/19.5%, respectively [5]. Among the studies that evaluated each type of ablation modality, the largest was conducted for RFA by de Baère et al. using their prospective database, which included 566 patients with 1037 tumors [12]. In their study, the 1-, 2-, 3-, 4- and 5-year OS/PFS rates after RFA were 92.4%/40.2%, 79.4%/23.3%, 67.7%/16.4%, 58.9%/13.1%, and 51.5% (the 5-year PFS was not available), respectively [12]. Other prospective or retrospective studies on RFA demonstrated comparable 3-year OS rates of approximately 50–75% [6, 10, 11, 15, 16], except for one prospective study by Wang et al., which showed a 3-year OS rate of 14% [13]. Wang et al. attributed the poor OS to a high proportion of patients with multiple lung metastases and extrathoracic diseases in their study cohort [13]. Vogl et al. conducted a prospective study on MWA that included 80 patients with 130 tumors and reported the 1- and 2-year OS rates to be 91.3% and 75.0%, respectively [14]. Callstrom et al. recently reported the results of their prospective study on CA, which included 128 patients with 224 tumors; the 1- and 2-year OS rates after CA were 97.6% and 86.6%, respectively [8]. Furthermore, de Baère et al. prospectively evaluated the long-term outcomes of CA in 40 patients with 60 tumors [7, 17]; the 3- and 5-year local tumor control rates were 87.9% and 79.2%, respectively, and the 3- and 5-year OS rates were 63.2% and 46.7%, respectively [7].

**Table 1:** Studies on ablative therapies for lung metastases from mixed types of primary tumor

Author	Publication year	Type of ablation	No. of patients	No. of tumors	Type of primary tumor	Follow-up [months]	Local tumor control [%]	Overall survival [months]		Overall survival [%] (progression-free survival [%])								
										1-year		2-year		3-year		4-year		5-year
Nguyenhuy et al. [5]	2022	RFA MWA CA	1804^a^	≥ 3344	CRC, sarcoma, RCC, etc	–	91 at 1 year	62 (median)		92.3 (53.1)		80.0 (34.8)		67.9 (25.4)		58.8 (22.2)		50.7 (19.5)
Tselikas et al. [6]	2021	RFA	126	223	CRC, RCC, sarcoma, etc	31 (median)	81.4 at 2 years	–	94 (38.9)		–		72 (14.8)		–		–	
de Baère et al. [7]	2021	CA	40	60	CRC, RCC, sarcoma, etc	–	79.2 at 5 years	–	97.5		84.3		63.2		–		46.7	
Callstrom et al. [8]	2020	CA	128	224	CRC, RCC, sarcoma, etc	–	77.2 at 2 years	–	97.6		86.6				–		–	
Nour-Eldin et al. [9]	2017	MWA	63	104	Bronchial, breast, RCC, etc	–	92.3 at 2 years	34.1 (mean)	97.6 (93.4)		79.9 (86.6)		62.3		45.4			
		RFA	29	49			79.6 at 2 years	34.8 (mean)	81.5 (89.4)		50.0 (68.2)		45.5		24.2		–	
		LITT	17	22			72.7 at 2 years	35.3 (mean)	93.8 (79.2)		56.3 (70.4)		50.0		31.3		–	
Fanucchi et al. [10]	2016	RFA	61	86	CRC, HNC, sarcoma, etc	28 (median)	–	65 (mean)	94.8		–		49.0		–		44.5	
Omae et al. [11]	2016	RFA	123	222	CRC, LC, HCC, etc	45.7 (median)	–	90 (median)	95 (58)		83 (41)		76 (33)		68 (28)		62 (25)	
de Baère et al. [12]	2015	RFA	566	1037	CRC, RCC, Sarcoma, etc	35.5 (median)	89.0 at 4 years	62 (median)	92.4 (40.2)		79.4 (23.3)		67.7 (16.4)		58.9 (13.1)		51.5	
Wang et al. [13]	2015	RFA	67	115	CRC, LC, sarcoma, etc	24 (median)	87.8	24 (median)	83.6 (55.7)		46.3		14.3		–		–	
Vogl et al. [14]	2011	MWA	80	130	CRC, breast, HCC, etc	6–24 (range)	73.1	–	91.3		75		–		–		–	
von Meyenfeldt et al. [15]	2011	RFA	46	90	CRC, sarcoma, RCC, etc	22 (median)	65 at 2 years	55 (median)	84 (33)		–		69 (11)		–		–	
Chua et al. [16]	2010	RFA	148	–	CRC, RCC, LC, etc	29 (median)	96	51 (median)	–		–		60		–		–	

*RFA* radiofrequency ablation, *MWA* microwave ablation, *CA* cryoablation, *LITT* laser interstitial thermal therapy, *CRC* colorectal cancer, *RCC* renal cell carcinoma, *Bronchial* bronchial carcinoma, *Breast* breast cancer, *HNC* head and neck cancer, *LC* lung cancer

^a^The whole study included 1804 patients with at least 3344 lung metastases in 23 studies, and the survival analysis was derived from 1660 patients in 22 studies

The above-mentioned studies tended to have relatively large study populations because they included a variety of primary tumors, allowing an overview of the general outcomes of thermal ablation therapies for lung metastases. However, survival outcomes might be affected by the proportion of each type of primary tumor included in each study. Indeed, the type of primary tumor is significantly correlated with survival in several studies [11–13]. Consequently, it is necessary to review the outcomes of ablative therapies for each type of tumor separately. Reportedly, prognostic factors other than the type of primary tumor include the presence of extrathoracic metastases [6], the size and number of lung metastases [6, 12], the response to treatment [14, 16], and the disease-free interval [12, 16]. Thus, the discrepancy between outcomes in each study might be caused by different patient conditions, including pre-ablation tumor burden.

### Outcomes by each type of primary tumor

#### Colorectal cancer

Table 2 shows the outcomes of ablative therapies for lung metastases from CRC [12, 18–31]. RFA was the most frequently used treatment in the listed studies. Although most studies had a retrospective design, Hasegawa et al. recently conducted a multicenter prospective trial in Japan [18]. In this trial, RFA was performed in 70 patients with 100 surgically resectable lung metastases (≤ 5 in number per patient and ≤ 3 cm in size) and an excellent 3-year OS rate of 84% was achieved [18]. Some studies reported the long-term outcomes of RFA based on relatively long follow-up periods. Zhong et al. retrospectively evaluated the outcomes of RFA in patients with histologically confirmed CRC lung metastases [19]. The 1-, 3-, 5-, 7-, and 9-years OS/PFS rates were 96.7%/66.7%, 74.7%/31.2%, 44.1%/25.9%, 27.5%/21.2%, and 16.3%/5.9%, respectively, with a median follow-up duration of 45.5 months [19]. Fonck et al. and Matsui et al. showed similar 5-year survival rates of 54.7% and 51.6%, respectively, in their retrospective studies [23, 27]. In a study on RFA for lung metastases from various primary tumors, de Baère et al. performed subgroup analyses for each group of patients with colon and rectal cancer and reported 5-year OS rates of 56.0% and 49.6% in the colon and rectum groups, respectively [12]. In the other studies, the 3- and 5-year OS rates after ablative therapies for lung metastases from CRC were 44–61% and 20–30%, respectively [21, 22, 24, 26, 28–31]. Local tumor control rates by ablative therapies were approximately 80–90% in most studies; notably, several studies published from 2018 onward achieved local control rates of 90% or higher [18, 19, 21, 23]. Because these studies included tumors of relatively small sizes (mean or median, 10–14 mm), their favorable local control rates might be attributable to appropriate tumor selection based on the knowledge that tumors of large sizes are a risk factor for local tumor progression [12]. To select candidates likely to receive survival benefits from lung ablation, it is necessary to determine the prognostic factors after ablation. In several studies, a high pre-ablation carcinoembryonic antigen (CEA) value was a negative prognostic factor after ablative therapies [18, 21, 27]. In addition, Huo et al. found that CEA density, i.e., pre-ablation CEA per volume of all lung metastases, was associated with OS [24]. In contrast, Ferguson et al. identified post-RFA CEA values as a significant prognostic factor as opposed to pre-RFA values [26]. The presence of extrapulmonary metastases was also a significant prognostic factor in several studies and was associated with poor OS [20, 27, 31] or chemotherapy-free survival [23]. Regarding the association between systemic chemotherapy and post-ablation survival, Hasegawa et al. revealed that the absence of chemotherapy before RFA was significantly associated with poor OS [18]. Chua et al. demonstrated that patients who received adjunct chemotherapy before or after RFA showed better OS than those who did not [31]. Kurilova et al. showed that three or more lines of pre-ablation chemotherapy with or without targeted therapy was a negative prognostic factor after MWA [21]. Other negative prognostic factors in the previous studies included a larger number of lung metastases [12, 19, 29], a larger tumor size [12, 20, 22], rectal rather than colon cancer [18], a shorter progression-free interval [19], a worse response to RFA [31], and a lack of repeated RFA [33].

**Table 2:** Studies on ablative therapies for colorectal lung metastases

Author	Publication year	Type of ablation	No. of patients	No. of tumors	Size of tumors [mm]	Follow-up [months]	Local tumor control [%]	Median overall survival [months]	Overall survival [%] (progression-free survival [%])				
									1-year	2-year	3-year	4-year	5-year
Hasegawa et al. [18]	2020	RFA	70	100	10 (mean)	57 (mean)	91 at 3 years	–	96 (57)	–	84 (41)	–	69 (37)
Zhong et al. [19]	2020	RFA	60	125	14 (mean)	45.5 (median)	90 at 4 years	52	96.7 (66.7)	–	74.7 (31.2)	–	44.1 (25.9)
Hiyoshi et al. [20]	2019	RFA	43	188	12 (median)	24.3 (median)	90.7 (per patient)	52.7	–	–	–	–	–
Kurilova et al. [21]	2018	MWA	50	90	10 (median)	25.6 (median)	90	58.6	94	82	61	–	–
Cheng et al. [22]	2018	MWA	32	48	≤ 3 cm: 71% 3-4 cm: 17% 4-6 cm: 12%	–	87.5 at 3 months	31	79.5	63.1	44.4	–	–
Fonck et al. [23]	2018	RFA^a^ MWA^a^ CA^a^	209	630	10 (median)	50 (median)	94.2	67.6	95 (33.1)	85.5 (18.4)	–	–	54.7 (11.2)
Huo et al. [24]	2017	RFA^b^ MWA^b^	182	–	–	27 (median)	–	33	92 (52)	–	46 (14)	–	30 (9)
Vogl et al. [25]	2016	MWA	47	103	5–50 (range)	–	88.3	32.8	82.7 (54.6)	67.5 (29.1)	(10.0)	16.6 (1.0)	–
		RFA	41	65	10–45 (range)	–	69.2	24.2	76.9 (77.3)	50.8 (50.2)	(30.8)	8.0 (16.4)	–
		LITT	21	25	8–42 (range)	–	68.0	22.1	95.2 (96.8)	47.6 (52.7)	(24.0)	23.8 (19.1)	–
de Baère et al. [12]	2015	RFA	Colon 191^c^	–	–	–	83.8 at 3 years	–	92.9 (37.6)	–	76.1 (17.0)	–	56.0 (14.8)
			Rectum 102^c^	–	–	–	69.3 at 3 years	–	93.6 (30.4)	–	64.9 (8.6)	–	49.6 (6.4)
Ferguson et al. [26]	2015	RFA	157	434	–	28 (mean)	88.5 (per procedure)	33.3	89	–	44	–	19.9
Matsui et al. [27]	2015	RFA	84	172	12 (median)	37.5 (median)	86.0	67.0	95.2	–	65.0	–	51.6
Gillams et al. [28]	2013	RFA	122	398	17 (mean)	–	81	41	–	–	57	–	–
Petre et al. [29]	2013	RFA	45	69	4–35 (range)	18 (median)	87	46	95	72	50	–	–
Yamauchi et al. [30]	2011	CA	24	55	13 (mean)	40 (median)	59 at 3 years	43	91	–	59.6	–	–
Chua et al. [31]	2010	RFA	100	–	–	23 (median)	–	36	87	66	50	–	30

*RFA* radiofrequency ablation, *MWA* microwave ablation, *CA* cryoablation, *LITT* laser interstitial thermal therapy

^a^RFA, MWA and CA were used in 96.9%, 2.2%, and 0.9% of procedures, respectively

^b^RFA and MWA were used for 87.4% and 12.6% of patients, respectively

^c^Subgroups of patients with colon and rectal cancer, respectively, in a study cohort including various types of primary tumors

Considering the favorable outcomes seen in several studies, image-guided ablation is a valid treatment for lung metastases from CRC [3]. The current National Comprehensive Cancer Network guidelines for colon cancer state that ablative therapies may be considered alone or in conjunction with surgical resection for resectable lung metastases [32].

#### Sarcoma

The main series of ablative therapies for lung metastases from sarcoma are summarized in Table 3 [12, 33–36]. RFA was always performed in the listed studies, except for the most recent one by Bourgouin et al., in which MWA and CA were used [33]. A relatively wide range of survival rates was demonstrated; the 1- and 3-year OS rates were 81–100% and 47–85%, respectively [12, 33–36]. The primary sites of sarcoma in those studies varied and included the bone, soft tissue, and parenchymal organs. Therefore, the difference in survival outcomes might be attributed to different patient backgrounds in each study. The local efficacy was consistent and favorable across studies: local control rates of 85–95% were achieved for tumors with a mean or median size of 9–14 mm [12, 33–36]. Regarding prognostic factors, Koelblinger et al. reported that a short disease-free interval (≤ 24 months) between the diagnosis of the primary lesion and lung metastases was significantly associated with inferior OS [35]. Sato et al. found that extrapulmonary metastasis, noncurative ablation, and a short disease-free interval (≤ 12 months) after ablation were negative prognostic factors [34]. The treatment of lung metastases from sarcoma by a combination of thermal ablation and surgery has also been investigated; Nakamura et al. treated 92 patients with soft tissue sarcoma with lung metastasectomy (*n* = 70), RFA (*n* = 13), or both (*n* = 9) and reported the 5-year post-metastatic disease-specific survival rate to be 52% [37]. Disease-specific survival rates at 3 years were 66%, 59.2%, and 66.7% in the surgery, RFA, and surgery + RFA groups, respectively [37].

**Table 3:** Studies on ablative therapies for sarcoma lung metastases

Author	Publication year	Type of ablation	No. of patients	No. of tumors	Size of tumors [mm]	Median follow-up [months]	Local tumor control [%]	Overall survival [months]	Overall survival [%] (progression-free survival [%])				
									1-year	2-year	3-year	4-year	5-year
Bourgouin et al. [33]	2022	MWA^a^ CA^a^	27	61	11 (median)	25	89	–	100	89	82	–	–
Sato et al. [34]	2017	RFA	46	144	13.5 (mean)	16.7	84.7	31.7 (median)	80.6	70.1	47.1	–	–
de Baère et al. [12]	2015	RFA	51^b^	–	–	–	91.7	–	94.1 (43.0)	–	58.0 (26.5)	–	41.5 (15.9)
Koelblinger et al. [35]	2014	RFA	22	55	9 (mean)	20	95	51 (mean)	100 (53)	94 (23)	85	–	–
Palussière et al. [36]	2011	RFA	29	47	9 (median)	50	89	–	92.2	–	65.2	–	–

*MWA* microwave ablation, *CA* cryoablation, *RFA* radiofrequency ablation

^a^MWA and CA were used for 34 metastases in 13 patients and 27 metastases in 14 patients, respectively

^b^A subgroup of sarcoma patients in a study cohort including various types of primary tumors

#### Hepatocellular carcinoma

Table 4 shows the results of recent studies on ablative therapies for lung metastases from hepatocellular carcinoma (HCC) [38–41]. The 1-, 3-, and 5-year OS rates after lung ablation were reported to be 73–89%, 30–70%, and 26–31%, respectively, with most patients being treated with RFA [38–41]. A lower number of lung metastases was a favorable prognostic factor in several studies [38, 40]. Specifically, Yuan et al. showed that patients with ≤ 2 tumors and unilateral metastases had better OS rates than those with > 2 tumors and bilateral metastases, respectively [38]. Li et al. also found that ≤ 3 lung metastases were a favorable prognostic factor of OS [40]. In addition, studies by both Li et al. and Hiraki et al. demonstrated that lower serum α-fetoprotein levels and the absence of viable or uncontrolled intrahepatic tumors were associated with better OS [40, 41]. Other positive prognostic factors included a maximum tumor diameter of ≤ 3 cm, Child–Pugh class A disease, the absence of liver cirrhosis, and the absence of hepatitis C virus infection [40, 41]. All the above-mentioned studies had a retrospective design and a relatively small study population (< 40 patients). Thus, further investigation with a large population size is necessary to validate the usefulness of ablation therapies for lung metastases from HCC.

**Table 4:** Studies on ablative therapies for HCC lung metastases

Author	Publication year	Type of ablation	No. of patients	No. of tumors	Size of tumors [mm]	Median follow-up [months]	Local tumor control [%]	Median overall survival [months]	Overall survival [%] (progression-free survival [%])				
									1-year	2-year	3-year	4-year	5-year
Yuan et al. [38]	2020	RFA^a^ MWA^a^ CA^a^	39	–	15 (median)	13.5	84.2 at 1 month (per patient)	–	79.8	–	58	–	30.9
Lassandro et al. [39]	2020	RFA	26	42	14 (mean)	–	–	–	88.5	69.8	69.8	34.9	26.2
Li et al. [40]	2012	RFA	29	68	19.3 (mean)	23	82.8 at 1 month (per patient)	21	73.4 (59.7)	41.1 (28.2)	30	–	–
Hiraki et al. [41]	2011	RFA	32	83	14 (mean)	20.5	92 at 3 years	37.7	87	57	57	–	–

*HCC* hepatocellular carcinoma, *RFA* radiofrequency ablation, *MWA* microwave ablation, *CA* cryoablation

^a^RFA, MWA, CA, and RFA + MWA were used in 22, 8, 7, and 2 patients, respectively

#### Renal cell carcinoma

Regarding lung metastases from renal cell carcinoma (RCC), Gonnet et al. retrospectively evaluated the outcomes of RFA in 53 patients with 100 tumors: a local efficacy of 91% and 1-, 3-, and 5-year OS rates of 94.0%, 74.5%, and 61.8%, respectively, were seen with a median follow-up of 60.8 months [42]. This was comparable to the outcome of the subgroup analysis in the study by de Baère et al., who demonstrated 1-, 3-, and 5-year OS rates of 95.5%, 73.5%, and 53.8%, respectively, after RFA in 68 patients with renal tumors [12]. Similarly, the RCC subgroup in a meta-analysis by Nguyenhuy et al. showed 1-, 3-, and 5-year OS rates of 93.0%, 71.7%, and 57.5%, respectively [5]. Concerning prognostic factors, Gonnet et al. found that the T3/T4 stage of primary RCC was associated with unfavorable survival [42].

#### Breast cancer

Thermal ablation for lung metastases from breast cancer was investigated by several researchers. Meng et al. reported the outcomes of MWA in 32 breast cancer patients with 46 tumors [43]. In their study, the 1-, 3-, and 5-year OS rates were 96.5%, 53.3%, and 17.8%, respectively, with local tumor progression observed in 10.9% of the ablated lesions [43]. Patients without extrapulmonary metastases showed a significantly longer median survival time (median of 24 months with extrapulmonary metastases vs. 36 months without, *p* = 0.005) [43]. Wang et al. investigated the outcomes of RFA for residual lung metastases from breast cancer after systemic chemotherapy [44]. Complete response was achieved in 59 of 67 lesions (88%) in 35 patients, and the 1-, 2-, and 3-year OS rates were 88.6%, 59.3%, and 42.8%, respectively [44]. Two or more lung metastases, a lesion diameter of > 2 cm, and coexisting liver metastases were associated with unfavorable OS [44].

#### Esophageal cancer

Two retrospective studies investigated RFA for lung metastases from esophageal cancer. Matsui et al. evaluated the outcomes of RFA in 21 patients: local tumor progression was observed in 8 of 31 tumors (25.8%), and the 1-, 2-, and 3-year OS rates after RFA were 85.7%, 54.8%, and 38.4%, respectively [45]. The presence of viable extrapulmonary recurrences was an unfavorable prognostic factor [45]. Baba et al. analyzed the outcomes of 10 patients who underwent RFA for 17 metastases [46]. In their study, the local control rate was 83% at 1 year and the 1- and 2-year OS rates were 77.8% and 62.2%, respectively [46].

#### Head and neck cancer

Bonichon et al. recently reported the results of a retrospective study on thermal ablation for lung metastases from thyroid cancer [47]. In their study, 47 patients with 107 tumors were treated with 56, 9, and 10 sessions of RFA, MWA, and CA, respectively, achieving a 5-year local control rate of 94.8% and 2- and 3-year OS rates of 93.0% and 79.0%, respectively [47]. The histological type of thyroid cancer was a prognostic factor [47]. Pan et al. retrospectively evaluated the outcomes of RFA for lung metastases from nasopharyngeal carcinoma: local tumor progression was found in 3 of 23 metastases (13%) in 10 patients [48]. A matched-pair analysis showed the median OS after the diagnosis of lung metastases was different between patients treated with and without lung RFA (77.1 months vs. 32.4 months, *p* = 0.009) [48].

### Ablation for lung oligometastases

The role of ablative therapies for lung oligometastases, i.e., a limited number of metastases confined to the lung, has been of particular interest to researchers. Because the presence of extrathoracic metastases and the number of metastases affect survival outcomes, patients with oligometastatic lung disease are theoretically assumed to be favorable candidates for lung ablation. In a meta-analysis by Nguyenhuy et al., a subgroup analysis of patients with lung oligometastases showed 1-, 3-, and 5-year OS rates of 96%, 76.4%, and 54%, respectively [5]. Tselikas et al. retrospectively reviewed patients with lung oligometastases (up to five in number with a maximum diameter of 4 cm and without pleural invasion or mediastinal lymph node metastases) from various types of primary tumors and reported 1- and 3-year OS rates of 94% and 72%, respectively (Table 1) [6]. Omae et al. evaluated the survival of 123 patients after RFA for lung oligometastases from various primary lesions, defined as up to five metastases confined to the lung with the primary lesion and other metastases eradicated at the time of initial RFA, and showed favorable 1-, 3-, and 5-year OS rates of 95%, 76%, and 62%, respectively (Table 1) [11]. The results of these studies suggest that thermal ablation is a promising treatment option for oligometastatic lung disease. Ablative therapies were preferentially used on patients with five or fewer lung metastases; however, the maximum number of metastases to be ablated has not been clearly defined and may depend on the growth rate of the tumors [3].

### Comparison of ablation modalities

In several studies, the treatment outcomes of different ablation modalities were compared. Nour-Eldin et al. reported that the local tumor progression rates after ablation for lung metastases from non-CRC were 7.7%, 20.4%, and 27.3% for MWA, RFA, and LITT, respectively (*p* = 0.012) (Table 1) [9]. MWA was beneficial for PFS (median 23.5, 19.9, and 16.7 months for MWA, LITT, and RFA, respectively, *p* = 0.048) [9]. Vogl et al. retrospectively compared the outcomes of MWA, RFA, and LITT for lung metastases from CRC (Table 2): MWA had the best local tumor control compared with RFA and LITT, although no difference in OS and PFS was observed between the three ablation methods [25]. They suggested MWA could create a wider ablation zone, thereby contributing to better local control [25]. However, the local control rate in their RFA cohort was 69%, which was low compared with other recent studies (Table 2). In a study on ablative therapies for lung metastases from HCC by Yuan et al., no difference in OS or local tumor PFS was found between ablation methods, including RFA, MWA, and CA [38]. Bourgouin et al. compared the local efficacy of MWA with that of CA for lung metastases from sarcoma (MWA for 34 tumors and CA for 27): no difference in local tumor control was detected [33]. In a meta-analysis that included primary and metastatic lung tumors, Yuan et al. performed subgroup analysis to compare the outcomes of RFA with that of MWA for metastatic lung tumors: RFA achieved a better median OS than did MWA (34.8 months vs. 18.7 months, *p* = 0.001) [49]. Nevertheless, they warned that this result should be interpreted with caution because the meta-analysis was conducted with only seven retrospective studies of RFA and one of MWA [49]. To date, RFA has been the most evaluated method among all thermal ablation therapies and is widely used to treat lung metastases. MWA has some theoretical advantages, such as a rapid temperature increase, a reduced heat-sink effect, and a larger ablation zone [3, 50]. Advantages of CA include preservation of collagenous architecture and less intraprocedural pain [3, 50]. However, compared with RFA, MWA and CA have less data on long-term outcomes available. Further data are needed to determine the most appropriate ablation modality.

## Conclusion

The outcomes of thermal ablation for lung metastases have been investigated in a growing number of studies over the past decade, with ablation for lung metastases from CRC being primarily evaluated. The present review revealed favorable local control and survival outcomes after ablative therapy for lung metastases from various types of primary tumors. The type of primary tumor and other background factors in each patient affect the prognosis; therefore, it is mandatory to take these factors into consideration when determining the indication for ablation. RFA has been the most evaluated technique, and further studies investigating the effectiveness of MWA and CA are warranted to allow the different ablation modalities to be compared thoroughly. In addition, despite not being the subject of this review, comparative data on thermal ablation with surgery and radiation therapy should also be accumulated to define the role of thermal ablation in the management of lung metastases.
